# Climate finance opportunities for health and health systems

**DOI:** 10.2471/BLT.23.290785

**Published:** 2024-02-29

**Authors:** Josephine Borghi, Soledad Cuevas Garcia-Dorado, Blanca Anton, Domenico Gerardo, Giulia Gasparri, Mark Hanson, Agnès Soucat, Flavia Bustreo, Etienne V Langlois

**Affiliations:** a London School of Hygiene & Tropical Medicine, Keppel Street, London WC1E 7HT, England.; b Instituto de Economía, Geografía y Demografía, Consejo Superior de Investigaciones Científicas, Madrid, Spain.; c Partnership for Maternal, Newborn and Child Health, World Health Organization, Geneva, Switzerland.; d Faculty of Medicine, University of Southampton, Southampton, England.; e Agence Française de Développement, Paris, France.; f Fondation Botnar, Basel, Switzerland.

## Abstract

Climate change poses significant risks to health and health systems, with the greatest impacts in low- and middle-income countries – which are least responsible for greenhouse gas emissions. The Conference of Parties 28 at the 2023 United Nations Climate Change Conference led to agreement on the need for holistic and equitable financing approaches to address the climate and health crisis. This paper provides an overview of existing climate finance mechanisms – that is, multilateral funds, voluntary market-based mechanisms, taxes, microlevies and adaptive social protection. We discuss these approaches’ potential use to promote health, generate additional health sector resources and enhance health system sustainability and resilience, and also explore implementation challenges. We suggest that public health practitioners, policy-makers and researchers seize the opportunity to leverage climate funding for better health and sustainable, climate-resilient health systems. Emphasizing the wider benefits of investing in health for the economy can help prioritize health within climate finance initiatives. Meaningful progress will require the global community acknowledging the underlying political economy challenges that have so far limited the potential of climate finance to address health goals. To address these challenges, we need to restructure financing institutions to empower communities at the frontline of the climate and health crisis and ensure their needs are met. Efforts from global and national level stakeholders should focus on mobilizing a wide range of funding sources, prioritizing co-design and accessibility of financing arrangements. These stakeholders should also invest in rigorous monitoring and evaluation of initiatives to ensure relevant health and well-being outcomes are addressed.

## Résumé

Le changement climatique fait peser des risques considérables sur la santé et les systèmes de santé, affectant principalement les pays à revenu faible et intermédiaire – alors qu'ils contribuent le moins aux émissions de gaz à effet de serre. Lors de la Conférence des Nations Unies sur le changement climatique de 2023, la 28^e^ Conférence des Parties a abouti à un accord sur la nécessité d'adopter des approches de financement équitables et holistiques pour résoudre la crise climatique et sanitaire. Le présent document offre un aperçu des dispositifs de financement climatique existants – à savoir des fonds multilatéraux, des mécanismes de marché volontaires, des micro-taxes et une protection sociale adaptative. Nous évoquons la possibilité de recourir à ces approches en vue de promouvoir la santé, de générer des ressources supplémentaires pour le secteur de la santé et de renforcer la viabilité et la résilience des systèmes de santé; nous nous intéressons également aux défis que représente leur mise en œuvre. Nous suggérons que les professionnels de la santé publique, les responsables politiques et les chercheurs profitent de cette occasion pour obtenir des fonds climatiques afin d'améliorer la santé et de développer des systèmes de santé durables et adaptés au changement climatique. Souligner tout l'intérêt, pour l'économie, d'investir dans la santé peut aider à inscrire la santé en priorité dans les initiatives de financement climatique. Réaliser des progrès significatifs implique que la communauté internationale prenne conscience des enjeux sous-jacents en matière d'économie politique, enjeux qui ont jusqu'à présent limité le potentiel du financement climatique dans l'atteinte des objectifs de santé. Pour y remédier, nous devons restructurer les institutions financières afin d'accroître l'autonomie des communautés en première ligne face à la crise climatique et sanitaire, et de faire en sorte que leurs besoins soient satisfaits. Les efforts des parties prenantes à l'échelle nationale et mondiale doivent porter sur la mobilisation d'un large éventail de sources de financement, en mettant l'accent sur la conception conjointe et l'accessibilité des modalités financières. Ces parties prenantes doivent en outre investir dans un suivi étroit et une évaluation rigoureuse des initiatives pour veiller à obtenir des résultats pertinents en termes de santé et de bien-être.

## Resumen

El cambio climático plantea riesgos importantes para la salud y los sistemas sanitarios, con mayores impactos en los países de ingresos bajos y medios, que son los menos responsables de las emisiones de gases de efecto invernadero. La 28.ª Conferencia de las Partes en la Conferencia de las Naciones Unidas sobre el Cambio Climático de 2023 condujo a un acuerdo sobre la necesidad de enfoques de financiación holísticos y equitativos para abordar la crisis climática y sanitaria. Este documento ofrece una visión general de los mecanismos de financiación climática existentes, es decir, los fondos multilaterales, los mecanismos voluntarios basados en el mercado, los impuestos, los microimpuestos y la protección social adaptable. Analizamos el uso potencial de estos enfoques para promover la salud, generar recursos adicionales para el sector sanitario y mejorar la sostenibilidad y la resiliencia de los sistemas sanitarios. Sugerimos que los profesionales de la salud pública, los responsables de formular las políticas y los investigadores aprovechen la oportunidad de utilizar la financiación climática para mejorar la salud y los sistemas sanitarios sostenibles y resilientes al cambio climático. Destacar los beneficios más amplios de invertir en salud para la economía puede ayudar a priorizar la salud dentro de las iniciativas de financiación climática. Para lograr avances significativos será necesario que la comunidad mundial reconozca los problemas de economía política subyacentes que hasta ahora han limitado el potencial de la financiación para abordar los objetivos de salud. Para superar estos desafíos, necesitamos reestructurar las instituciones financieras para empoderar a las comunidades que se encuentran en primera línea de la crisis climática y sanitaria y asegurar que se satisfacen sus necesidades. Los esfuerzos de las partes interesadas a nivel mundial y nacional deben centrarse en movilizar una gran variedad de fuentes de financiación y priorizar el diseño conjunto y la accesibilidad de los acuerdos de financiación. Estas partes interesadas también deben invertir en la supervisión y evaluación rigurosas de las iniciativas para garantizar que se abordan los resultados pertinentes en materia de salud y bienestar.

## ملخص

يمثل التغير في المناخ مخاطر ملموسة على الصحة والأنظمة الصحية، حيث تكون أكبر التأثيرات في الدول ذات الدخل المنخفض والدخل المرتفع، وهي الأقل مسؤولية عن انبعاثات غازات الاحتباس الحراري. وأدى اجتماع الأطراف رقم 28 في مؤتمر الأمم المتحدة لتغير المناخ لعام 2023، إلى الاتفاق على الحاجة إلى أساليب تمويلية شاملة وعادلة لمعالجة أزمة المناخ والصحة. تقدم هذه الورقة لمحة عامة عن آليات تمويل المناخ الحالية، أي الصناديق متعددة الأطراف، والآليات التطوعية المعتمدة على السوق، والضرائب، والرسوم البسيطة، والحماية الاجتماعية التكيفية. نحن نناقش الاستخدام المحتمل لهذه الأساليب لتعزيز الصحة، واستحداث موارد إضافية للقطاع الصحي، وتعزيز استدامة النظام الصحي ومرونته، ونستكشف أيضًا تحديات التنفيذ. نحن نقترح أن ينتهز الممارسون في الصحة العامة، وواضعو السياسات، والباحثون الفرصة للاستفادة من التمويل المناخي من أجل صحة أفضل، وأنظمة صحية مستدامة وقادرة على التكيف مع المناخ. إن التأكيد على الفوائد الأوسع للاستثمار في الصحة بالنسبة للاقتصاد، يمكنه أن يساعد في منح الأولوية للصحة ضمن مبادرات تمويل المناخ. وسيتطلب تحقيق تقدم ملموس، اعتراف المجتمع العالمي بالتحديات الاقتصادية السياسية، والتي قللت حتى الآن من قدرة تمويل المناخ على تحقيق الأهداف الصحية. ولمواجهة هذه التحديات، نحتاج إلى إعادة هيكلة مؤسسات التمويل لتمكين المجتمعات التي توجد في مقدمة المواجهة مع أزمة المناخ والصحة وضمان الوفاء باحتياجاتها. يجب تركيز جهود أصحاب المصلحة على المستوى العالمي والوطني على تعبئة مجموعة واسعة من مصادر التمويل، ومنح أولوية التصميم المشترك وإمكانية الوصول إلى ترتيبات التمويل. وينبغي لمثل هؤلاء أصحاب المصلحة الاستثمار في كل من المراقبة والتقييم بشكل جاد للمبادرات، لضمان التعامل مع النتائج الصحية والرفاهية ذات الصلة.

## 摘要

气候变化对人类健康和卫生系统有着重大影响风险,对温室气体排放量最小的低收入和中等收入国家的影响最大。2023 年联合国气候变化会议第 28 次缔约方大会就应对气候和健康危机而需采取全面和公平的融资方法达成了一致。本文概述了目前已建立的气候金融机制,包括多边基金、基于市场的自愿减排机制、税收、微税和适应性社会保护。我们讨论了将这些方法用于促进健康、为卫生部门提供额外资源和增强卫生系统可持续性和复原力的可能性,并探讨了实施这些方法所面临的挑战。我们建议公共卫生从业者、政策制定者和研究人员抓住机会,利用气候融资打造更健康和可持续的、适应气候变化的卫生系统。强调了卫生投资对经济的更广泛益处,这有助于在气候融资倡议中优先考虑卫生问题。如要取得有意义的进展,则需要国际社会承认迄今为止可能阻碍气候融资实现卫生目标的潜在政治经济挑战。为了应对这些挑战,我们需要重组融资机构,增强受到气候变化影响最为显著的地区的权能,并确保满足他们的需求。全球层面和国家层面的利益相关者的工作应侧重通过多种方式筹集资金,优先考虑共同制定融资计划及其可及性。这些利益相关者还应对计划的严格监管和评估进行投资,以确保实现相关健康和福祉成果

## Резюме

Изменение климата подвергает значительным рискам здоровье и системы здравоохранения, оказывая наибольшее воздействие на страны с низким и средним уровнем дохода, которые в наименьшей степени ответственны за выбросы парниковых газов. На 28-й Конференции участвующих сторон в рамках Конференции ООН по изменению климата в 2023 году было достигнуто соглашение о необходимости применения целостных и справедливых подходов к финансированию для решения проблемы климатического кризиса и кризиса в области здравоохранения. В данном документе представлен обзор существующих механизмов финансирования климатических программ: многосторонних фондов, добровольных рыночных механизмов, налогов, микросборов и адаптивной социальной защиты. В статье рассматриваются потенциальные возможности использования этих подходов для укрепления здоровья, привлечения дополнительных ресурсов в сектор здравоохранения и повышения устойчивости и жизнеспособности системы здравоохранения, а также изучаются проблемы их реализации. Авторы предлагают практикующим специалистам в области общественного здравоохранения, ответственным лицам и исследователям воспользоваться возможностью и привлечь средства, выделяемые на борьбу с изменением климата, для улучшения здоровья и создания устойчивых к изменению климата систем здравоохранения. Акцент на более широких преимуществах инвестиций в здравоохранение для экономики может содействовать определению приоритетности здравоохранения в рамках инициатив по финансированию климатически значимой деятельности. Для достижения значительного прогресса необходимо, чтобы мировое сообщество признало основные политико-экономические проблемы, которые до сих пор ограничивали потенциал финансирования климатически значимой деятельности для достижения целей в области здравоохранения. Для решения этих проблем необходимо изменить структуру финансовых институтов с целью расширения прав и возможностей сообществ, находящихся на переднем крае кризиса в области климата и здравоохранения, и обеспечения удовлетворения их потребностей. Необходимо направить усилия заинтересованных сторон на глобальном и национальном уровнях на мобилизацию широкого спектра источников финансирования, уделяя приоритетное внимание совместной разработке и доступности финансовых механизмов. Кроме того, этим заинтересованным сторонам следует вкладывать средства в тщательный мониторинг и оценку инициатив, чтобы обеспечить достижение соответствующих результатов в области здравоохранения и благосостояния.

## Introduction

An estimated 3.6 billion people worldwide are highly vulnerable to climate change and therefore exposed to additional health risks. Climate change is expected to contribute 250 000 additional deaths per year by 2030.^1^ Climate change also affects economic growth,^2^ which can alter governments’ resource allocations to health. The distribution of the climate-health burden is highly inequitable, with the greatest impacts in low-and middle-income countries. Paradoxically, these countries account for less than 4% of the global greenhouse gas emissions.^3^

Governments have recognized the interconnected pathways between climate change and health, leading to consensus on bolstering intersectoral collaboration and investing in greener, more resilient health systems, as recognized in the Conference of Parties 28 (COP28) United Arab Emirates (UAE) Declaration on Climate and Health.^4^ Moreover, at COP28 during the United Nations Climate Change Conference in December 2023, multilateral development banks and funders pledged a total of 1 billion United States dollars (US$) to address the climate-health crisis, and agreed on guiding principles for financing climate and health solutions.^5^ These principles promote holistic and equitable financing approaches that embed health and climate goals.

In this paper, we provide an overview of existing climate finance mechanisms and their use to promote health, generate additional health sector resources, and enhance health system sustainability and resilience, and we explore implementation challenges. By climate finance we refer to finance activities and instruments that are used for either of two main activities. First, climate change mitigation, including reducing carbon emissions. Second, adaptation initiatives such as investments to reduce vulnerability to climate change and build resilience, or to address climate loss and damage – that is, the consequences of climate change that go beyond what people can adapt to. In Box 1, we have defined the key terms used in this paper.

Box 1 **Key terms used in the paper**

**Climate loss and damage**

Refers to the consequences of climate change that go beyond the capacity of communities to adapt, including those resulting from catastrophic weather events, droughts, sea-level rise, ocean acidification and glacial retreat.

**Non-economic loss and damage**

Include the loss of human lives, trauma, displacement and loss of ways of life and cultures. Human health impacts are considered a key non-economic damage in the context of climate loss and damage.^10^

**Carbon credits and offsets**

A carbon credit is a token representing the avoidance or removal of greenhouse gas emissions, measured in tonnes of carbon dioxide equivalent.^7^ Offsetting is the act of compensating for CO_2_ emissions by purchasing carbon credits in voluntary markets. Intermediaries and brokers play an important role in carbon transactions, potentially capturing an important proportion of rents in the market, as well as leading to substantial lack of transparency.^8^ Carbon credits and carbon offsets are often used interchangeably.

**Microlevies**

Involve the voluntary subscription of organizations or countries to earmark funding for specific sector development activities, including health.^11^

**Carbon pricing**

Direct carbon pricing includes carbon taxation and emissions trading schemes (similar to offsetting, but where companies and firms are legally required to purchase permits to cover their emissions) as well as voluntary offsets. Indirect carbon pricing includes the removal of fossil fuel subsidies and fossil fuel taxation, which might not explicitly tax carbon emissions but can generate equivalent incentives.^9^

**Adaptive social protection**

When safety nets, cash transfers and insurance schemes are targeted to climate risks, also offering an opportunity to help mitigate the health consequences of climate hazards.^8^

**Nationally determined contributions**

Countries’ self-defined national climate pledges under the Paris Agreement, detailing what they will do to help meet the global goal to pursue 1.5 °C, adapt to climate impacts and ensure sufficient finance to support these efforts.^6^

CO_2_: carbon dioxide.

## Multilateral funds

Multilateral funds can support mitigation initiatives or adaptation strategies. Such funds offer an important opportunity for health and health systems, as they allow for the inclusion of health and well-being goals.^12^^,^^13^ Some of the larger multilateral funds include the Green Climate Fund, Adaptation Fund and Global Environment Facility. Public Development Banks have related funds such as the Clean Technology Fund, and others linked to deforestation, smallholder agriculture or renewable energies. However, a recent study estimates that of the US$ 9093 million of multilateral adaptation finance support, only US$ 522 million (5.7%) went to health-related activities between 2009 and 2019.^14^ This sum is much less relative to other sectors such as energy, transport or agriculture.^13^ These health-related funds are also mainly in the form of grants. To increase resource availability in the health sector, financial institutions could, in addition to providing grants, channel loans, equity or guarantees to climate-related action.^15^

One of the factors limiting the uptake of such funding by the health sector is the limited number of accredited health institutions that can apply for climate finance.^13^ Accredited organizations work with countries to develop project ideas and submit funding proposals for approval to multilateral climate funds.^16^ Organizations can be public, private or nongovernmental as long as they meet the accreditation standards of the multilateral climate funds, which include, for example, capacity for financial management. Multilateral climate funds and the World Health Organization (WHO) are committed to enhancing access to funds and accreditation for health institutions.^17^ However, efforts are also needed to ensure that funds are targeted to countries with both the greatest economic needs and climate vulnerability, and to increase the focus of investments on adaptation. Currently, low-income countries receive only one fifth of approved funds across all sectors, predominantly for mitigation efforts.^18^ The Green Climate Fund recently approved a US$ 25 million project in Lao People's Democratic Republic, to strengthen health and climate early warning systems and build health system and community resilience across the country. This project will provide useful learning lessons for future initiatives. Capacity-building at the country level will also be important to ensure climate and health expertise is available to support funding applications.

The newly created multilateral Climate Loss and Damage Fund provides an important opportunity for the health sector. Health is considered a key non-economic loss and damage,^10^ which would enable the fund to address health impacts of climate hazards, including the needs of displaced populations, and rebuild damaged health infrastructure. Research valuing health-related loss and damages from climate change will be critical to ensure that the fund fully accounts for these and can support health-related needs. However, many uncertainties remain regarding the fund’s operationalization,^19^ affecting its actual potential for addressing health and other non-economic loss and damages. Uncertainties remain about the volume of available funds (given the voluntary nature of contributions), the role of the World Bank as host and the fund’s degree of reliance on concessional loans.^20^

## Voluntary market-based mechanisms

Carbon credits are also used to finance multisectoral activities or interventions with substantial health impacts, such as investments in clean cookstoves,^21^^,^^22^ water treatment projects^23^ or community forest fire management. Carbon credits represent the avoidance of one tonne of carbon dioxide equivalent with respect to a hypothetical counterfactual. Emissions avoided because of specific projects can be verified against existing standards and sold to third parties wanting to compensate for their own emissions. Carbon credits and voluntary offsetting have the potential to mobilize private sector funds, constituting a potential source of funds for climate and health. However, the volatility and lack of transparency in carbon markets, as well the controversy around their effectiveness as an emission reduction strategy, constitute important concerns for the use of carbon credits to finance health.^24^ Additionally, existing experiences show that it can be complex to align health and climate goals. For example, the cookstoves and fuel switches that most enhance health outcomes do not necessarily maximize emission reduction.^21^^,^^22^ Equally, households may switch from water boiling towards a less safe treatment to obtain carbon credit income, with negative health impacts.^23^ Careful project design, implementation and monitoring, and consideration of health goals from the outset are crucial in light of these concerns.^19^^,^^20^

## Taxes and microlevies

Taxation and microlevies aimed at mitigation at the global or national levels may also support the mobilization of revenues for the health sector alongside emissions reductions. The main distinction between taxes and microlevies is the degree of obligation of contributions. Taxes refer to obligatory charges on activities associated with carbon emissions, and microlevies involve the voluntary subscription of organizations or countries to earmarking revenue to benefit development activities such as health.

Microlevies on airline tickets, and oil and gas revenues are relevant examples. The airline tax, introduced in 2006, taxes the profits from airline tickets to fund global health programmes, mainly through Unitaid.^25^ Another example includes the United Nations Trust Fund to Prevent Chronic Malnutrition, whereby 0.1% of mining, gold, oil and gas revenues of subscribing countries are deposited into the fund.^25^

While seen as predictable and offering long-term funding, microlevies account for a limited amount of revenue and are vulnerable to economic fluctuations.^11^ Channelling these funds to health also requires earmarking for health and creating a global pooling mechanism, with an inclusive governance and effective country implementation mechanism. Furthermore, global taxation and microlevies can require many years of negotiations, resulting in additional administration costs and continuing dependency of low- and middle-income countries on higher-income countries’ revenue and/or aid.^25^ Investments in carbon pricing initiatives at the national level are therefore equally important.

Carbon pricing, through emissions trading schemes and carbon taxes, can reduce pollution from fossil fuels, promote health and generate government revenue that can benefit the health sector if it is earmarked for health.^26^ Eliminating fossil fuel subsidies, which in 2022 accounted for a record US$ 1.3 trillion, has the potential to create fiscal space that can be redirected to health^27^^,^^28^ and can lead to health co-benefits, potentially reducing health-care demand. Carbon pricing has been implemented in over 60 countries.^24^ There are a number of examples of carbon pricing revenue being channelled to the health sector, including Switzerland.^24^ Positive examples of revenue from fossil fuel subsidy removal benefiting the health sector can be found in a range of low- and middle-income countries^29^. The substantial literature on the political economy of carbon pricing revenue identifies some opportunities as well as challenges that apply to health-related uses. The earmarking of revenues for health could enhance the social acceptability of carbon pricing initiatives, facilitating their uptake.^30^ However, concerns remain regarding revenue instability and perverse incentives for emission reduction in the longer term.^31^

## Adaptive social protection

While not specifically climate finance, social protection – including safety nets, cash transfers and insurance schemes – can be targeted to climate risks.^8^ Adaptive social protection offers an opportunity to help reduce the health consequences of climate hazards, providing health-care access for climate-induced displaced populations, and encouraging post-hazard care uptake.^32^^,^^33^ Social protection schemes can also incentivize mitigation actions at the household level, such as installing solar panels or insulating houses, with health co-benefits.^31^ Perhaps most importantly for health, adaptive social protection mechanisms can greatly vary in terms of equity of outcomes and fund additionality, with experts cautioning against an excessive reliance on private insurance mechanisms.^34^

## Conclusion

Recent developments in international climate governance, including the new pledges announced at COP28 to address the climate-health crisis, represent an opportunity for engagement on the health and climate financing nexus.^35^ Public health practitioners, policy-makers and researchers should seize this opportunity to leverage climate funding for better health and sustainable, climate-resilient health systems.

However, meaningful progress will require the global community acknowledging and addressing the underlying political economy challenges that have so far limited the potential of climate finance to address health goals. Challenges include issues of geographical distribution of power within multilateral climate institutions; the degree of reliance on profit-generating and market-based financing mechanisms such as private climate insurance and carbon credits; the role of concessional loans in new loss and damage funding; and sector siloed decision-making and resource allocation.^27^

Efforts from global and national level stakeholders should focus on mobilizing a wide range of funding sources, prioritizing co-design and accessibility of financing arrangements, and investing in rigorous monitoring and evaluation of initiatives to ensure relevant health and well-being outcomes are addressed. To ensure climate finance supports health goals, these goals should be included in climate policies such as nationally determined contributions. Currently, only a small number of governments do so,^36^ although the rate is higher among low- and middle-income countries and those affected by climate-related extreme weather.^37^ Strengthening climate and health expertise will also be important to design effective financing arrangements that benefit joint goals. Emphasizing the wider benefits of investing in health for the economy can help prioritize health within climate finance initiatives.^38^ At the same time, it is imperative to work towards (re)structuring financing institutions to empower communities at the frontline of the climate and health crisis, including women, children and young people, and those in low-income countries to ensure their needs are met.
